# Using Machine Translation and Post-Editing in the TRAPD Approach: Effects on the Quality of Translated Survey Texts

**DOI:** 10.1093/poq/nfad060

**Published:** 2024-03-28

**Authors:** Diana Zavala-Rojas, Dorothée Behr, Brita Dorer, Danielly Sorato, Veronika Keck

**Affiliations:** Principal Investigator of the European Social Survey ERIC, Universitat Pompeu Fabra, Barcelona, Spain; and Deputy Director of the Research and Expertise Centre, Survey Methodology in the Political and Social Sciences Department, Universitat Pompeu Fabra, Barcelona, Spain; Head of Team, Cross-Cultural Survey Methods, Survey Design and Methodology Department, GESIS—Leibniz Institute for the Social Sciences, Mannheim, Germany; Head of the Translation Workpackage, European Social Survey ERIC, Mannheim, Germany; and Senior Researcher, Survey Design and Methodology Department, GESIS—Leibniz Institute for the Social Sciences, Mannheim, Germany; Researcher, Research and Expertise Centre for Survey Methodology, Political and Social Sciences Department, Universitat Pompeu Fabra, Barcelona, Spain; and PhD Candidate, Department of Translation and Language Sciences, Universitat Pompeu Fabra, Barcelona, Spain; Senior Client Training Consultant, The Nielsen Company (Germany) GmbH (NielsenIQ), Frankfurt am Main, Germany

## Abstract

A highly controlled experimental setting using a sample of questions from the European Social Survey (ESS) and European Values Study (EVS) was used to test the effects of integrating machine translation and post-editing into the Translation, Review, Adjudication, Pretesting, and Documentation (TRAPD) approach in survey translation. Four experiments were conducted in total, two concerning the language pair English-German and two in the language pair English-Russian. The overall results of this study are positive for integrating machine translation and post-editing into the TRAPD process, when translating survey questionnaires. The experiments show evidence that in German and Russian languages and for a sample of ESS and EVS survey questions, the effect of integrating machine translation and post-editing on the quality of the review outputs—with quality understood as texts output with the fewest errors possible—can hardly be distinguished from the quality that derives from the setting with human translations only.

## Introduction

Given its large impact on data quality in cross-national studies (Harkness et al. 2010), survey translation, that is, the translation and translation assessment of survey questionnaires, has consolidated as an important area of comparative survey methodology. The Translation, Review, Adjudication, Pretesting, and Documentation (TRAPD) approach to survey translation serves as a methodological gold standard (Harkness 2003), and variants of it are used to translate questionnaires in major multilingual projects such as the European Social Survey (European Social Survey 2024) (ESS), the Eurofound surveys (Eurofound 2017), and the European Values Study (Przepiórkowska and Behr 2017) (EVS). TRAPD has also been applied to the translation of questionnaires in medical and health research (Forsyth et al. 2007) or in market research (Kietzmann et al. 2016; Sha and Lai 2016).

Following the TRAPD method, at the “translation” stage (T in the acronym) two translators produce independent and parallel translations of the source questionnaire into a target language; or the source questionnaire is split among the translators. At least one of the translators is recommended to be a trained and/or professional translator or a linguist, while the second translator may be a social scientist. In a review meeting (R), the translations are discussed by the translators together with a reviewer; at the adjudication stage (A), an adjudicator is responsible for the final decisions on different translation options. Oftentimes, the roles of reviewer and adjudicator, both typically having a background in social sciences, are merged. The translated questionnaire is pretested before fieldwork (P) and the whole process is documented (D), including information on difficult translations, needed deviations, or remaining challenges in the translation.

Team members should combine survey knowledge, translation expertise, knowledge of the culture where the questionnaire will be administered, and knowledge related to the topic of the survey. While parallel translations offer variants to compare, the team-based discussion is at the heart of TRAPD, revisiting the decision-making process for the selection of wording as well as fostering interdisciplinary collaboration between professional translators and social scientists; after all, versions can be discussed taking into account different viewpoints by the different experts (Harkness 2003).

Until recently, the use of machine translation (MT) for survey translation has been discouraged in best-practice guidelines for cross-national survey methodology (Mohler et al. 2016b), so the T step was based on all human translations. Given the importance of survey translation quality in comparative studies, highly defective machine translations—as was still common some years ago—were suggesting neither a higher translation quality nor a more efficient translation process. However, the analysis of natural language by computational means has rapidly evolved in the last decade. And with it, MT quality has substantially improved after the development of artificial neural-network-based engines, neural MT (Way 2020; Nitzke and Hansen-Schirra 2021). Moreover, the availability of online machine translation tools, such as Google Translate and DeepL Translate, has allowed it to become a broadly used internet-based service. Given this availability, survey projects teams may perceive that MT has become a suitable method for questionnaire translation. From the angle of survey methodology, testing for the impact of new procedures before adopting them is fundamental to maintain data quality, as all potential sources of error should be considered, as well as their potential interactions (Smith 2011).

This experimental study integrates MT into the TRAPD approach and tests for its potential effects on translated survey questions. MT will be followed up by post-editing (PE), which is understood as the revision of raw machine translation output (more on this in the Methods section). We implemented a highly controlled experimental TRAPD approach to prevent process-related effects from confounding the comparison of the groups using MT against those using only human translation. In the TRAPD implementation reported here, the review and adjudication steps were merged. For this merged step, we will use the term review/er throughout the text. A difference in this study with respect to the theoretical approach of TRAPD is that we did not pretest our translations.

The main research question in this study is: Would replacing one of the initial human translations at the T step through MT and PE impact on the quality of the review output? If so, are the effects negative or positive for the outputs’ quality? The review output is the translation version resulting from the review discussion. Harkness and other proponents of the TRAPD model argued that translation quality is ensured in the review meeting in which the team discusses the translation options (Harkness, Pennell, and Schoua-Glusberg 2004; Harkness, Villar, and Edwards 2010; Mohler et al. 2016a); therefore, our experiments focus on assessing the effects of MT and PE on the output texts after these review meetings. Our secondary research questions explore group dependencies of the effects of MT and PE: Are effects conditional on the use of full or light PE? And, are Russian and German translations affected differently by MT and PE? These and the stakes of this research will be explained in detail in the next sections. Previewing the outcome, the overall results for integrating MT and PE into TRAPD are positive. Only in the German light-PE treatment group do we see a slightly higher error level compared to the control group, which may be negligible, though.

To the best of our knowledge, there have been no recommendations for the use of MT and PE in the field of academic social sciences surveys. At the same time, we acknowledge that neural MT is slowly entering the field of measurement instruments and is undergoing various tests of applicability also by other researchers (Iwai et al. 2019; Mondal, Mondal, and Mondal 2019).

### Machine Translation and Post-Editing

MT is a highly interdisciplinary scientific area, bringing together, among others, linguists, computer scientists, and translation scholars. Until recently, the language-service-provider community’s acceptance of MT was low; acceptance has become more widespread since the emergence of neural MT (Bahdanau, Cho, and Bengio 2015; Moorkens et al. 2018).

Findings from the European Language Industry Survey (2020) (ELIA et al. 2020) show that MT is the main technology trend for the future. Using MT is deemed to speed up the translation process, thus increasing translation volume as well as turnover (Koponen 2016). Texts obtained by MT are considered pretranslations because of subsequent revisions typically needed by a human (Sin-Wai 2017).

In translation practice, machine translation is commonly implemented in combination with post-editing. We distinguish between full and light PE. Full PE covers the production of accurate, comprehensible, and linguistically correct output that is similar or equal to human translation quality. Light PE, being a time-saving procedure, implies producing an output that is accurate and comprehensible, but not necessarily stylistically or grammatically adequate (Massardo et al. 2016). Full PE can be qualitatively as good as a human translation or even better (Daems et al. 2017), even though it can also show deficiencies compared to a human translation (Moorkens et al. 2018; Toral 2019). But would integrating a suboptimal light-PE version into a team review discussion be sufficient to arrive at a good review output? Given these two types of PE, this study tested whether their differentiation would make a difference on the quality of the review output.

MT is not suitable (at the time of writing) for all text types. The usability of MT heavily depends on the text corpora^1^ that are used for training MT engines (Nitzke and Hansen-Schirra 2021). Online MT tools have not been specifically trained for questionnaires or survey speak. Questionnaires are wording sensitive, and small wording differences may make a difference for respondents’ understanding and survey responding. Thus, we wanted to explore whether MT combined with different forms of PE can be used for the text-type survey questionnaires. The fact that literary translation, another wording-sensitive translation area, is exploring MT and PE (Moorkens et al. 2018) shows that the time is ripe to test MT and PE for survey translation.

Besides text type, the language pair used in machine translation is known to affect translation quality. Research has looked into MT quality for different language combinations (Doherty and O’Brien 2014; Castilho et al. 2017; Popović 2018), as the more distant languages are from each other, the more difficult they tend to be for MT. Would using different language combinations, in our study English-German and English-Russian, lead to different translation quality in the different experimental groups?

### Translation Quality and Translation Quality Assessment

Translation quality (TQ) can be approached both from the angle of the process and from the angle of the product (Gouadec 2010). Focusing in the following on the latter one, there is agreement that translation quality assessment is marked by complexity. There is almost never only one correct translation, and what counts as good, acceptable, or unacceptable depends on various factors, such as the text type, the intended purpose of a text, the target population, and further project-related specifications.

For assessing TQ, there are different approaches. Some test the texts on the target population that is intended to understand such texts. In this research, we evaluate the quality of the texts themselves. For the approaches analyzing the texts directly, one can differentiate between holistic and analytic approaches to TQ: holistic approaches evaluate the text as a whole, whereas analytic approaches allow for identifying specific translation problems and assessing the translation in its details (Lommel 2018, p. 122). For analytic quality assessments, error typologies are used (Castilho et al. 2018). These typologies include errors pertaining to domains such as adequacy and fluency, and allow for the possibility to weigh errors according to a severity level. Such models are typically applied by humans; this process is not error-free since subjective judgement can differ, for instance, as to whether an error counts as a lexical or a terminological error or how severe it is.

Clear guidelines, training, and regular practice with an error typology are practices to reduce the impact of subjectivity on error assignment (Saldanha and O’Brien 2014). Error coding schemes are often applied by two humans, enabling a systematic and comparative evaluation. Latest error typologies, such as the Multidimensional Quality Metrics (MQM), are deliberately comprehensive, applicable to both human and machine translation, and can be tailored to an actual project by selecting relevant subsets of errors (Castilho et al. 2018). MQM also represents and integrates most of the other commonly used translation error taxonomies in the industry (Vardaro, Schaeffer, and Hansen-Schirra 2019, p. 7). As such, MQM will be our method of choice for error coding. Alternative approaches include adequacy or fluency ratings as well as ranking of translations (Castilho et al. 2018), but these would not have allowed in-depth investigation of concrete translation problems occurring in the wording of the review outputs.

A further differentiation of translation quality is whether it is evaluated manually, that is, by humans, or by using algorithms. Algorithms produce metrics that compare source and (human- or machine-) translated target texts, such as the Bilingual Evaluation Understudy, or they run automated checks on specific matters, such as grammar, or other error categories (Castilho et al. 2018).

However, in general, while automated TQ checks are faster, cheaper, and more objective, many of them require a reference translation to evaluate the quality of the human and MT outputs. In addition, instead of having an evaluation of a whole sentence, as is common when using algorithms, our focus was also to assess the translations for errors in individual words, compound words, and phrases. Hence, in our study, we will not be using algorithms to assess TQ.

Moving from error coding as used in translation research and industry to cross-cultural survey methodology, TQ for survey translations is understood as retaining functional equivalence to the source, to the format of a source question as well as to its measurement properties; moreover, it requires adhering to the linguistic needs of the target language, and overall maintaining the same stimulus as the source (Harkness, Villar, and Edwards 2010). Equivalence is assessed by testing the translated texts among the target group, for instance, by cognitive pretests or pilot surveys (Harkness, Pennell, and Schoua-Glusberg 2004).^2^ However, the focus of this study was to evaluate the texts directly, their linguistic content. Thus, we analyze TQ and compare the text outputs in the experiments based on an error typology. We assume that there is an inverse relationship between errors and quality, in which texts presenting fewer errors are of higher quality, and on this basis we compare the errors between the different translated texts, in the concrete case, the review outputs.

## Method

### Design of the Experiments

**Figure 1.**
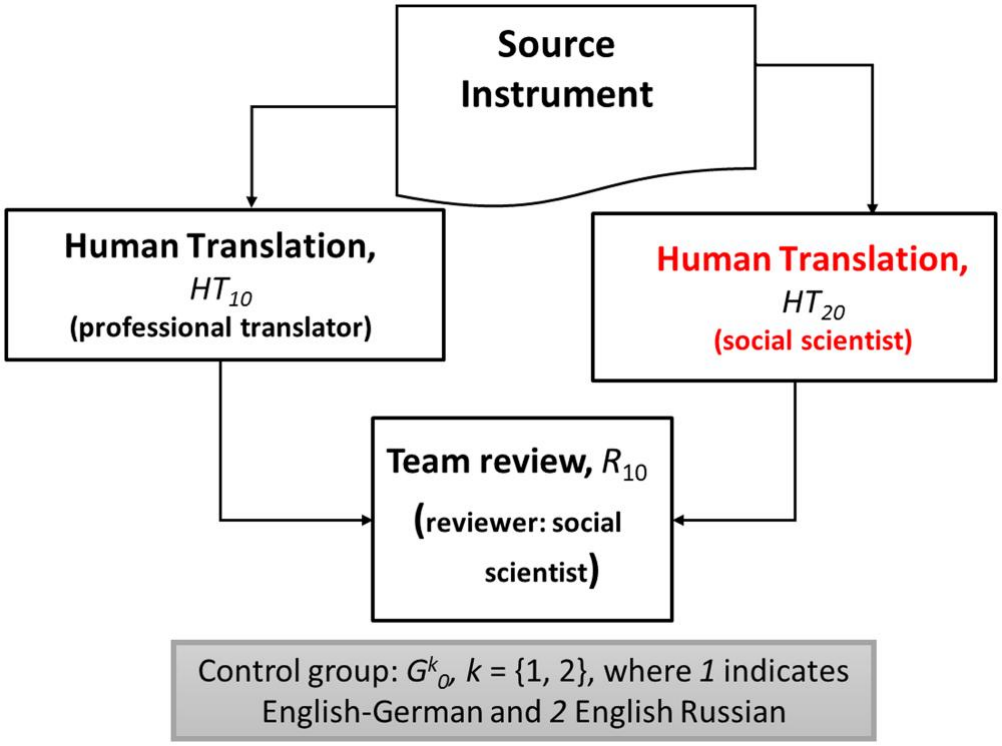
Summary of the control group.

**Figure 2.**
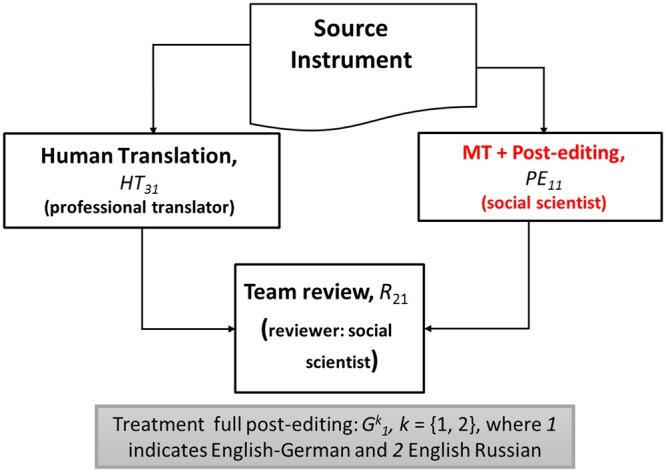
Summary of the treatment using full post-editing.

**Figure 3.**
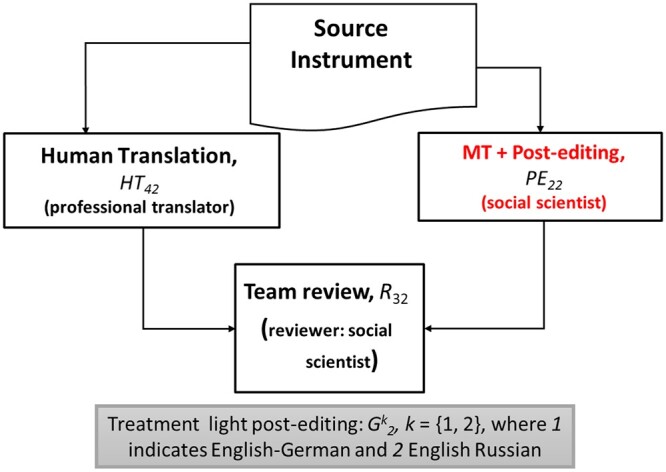
Summary of the treatment using light post-editing.

We designed and conducted four experiments to test for the impact of MT and PE in the review outputs produced in the TRAPD method. Figures 1–3 illustrate the design of the experiments.

The experiments are characterized by Equations 1 to 3. A language-specific control group, $G_{0}^{k}$, implements the translation step of the TRAPD without using machine translation at all. The initial translations are discussed in a team meeting at the review step where decisions are made about translation options and where the final translation, the review output, is produced. The process is documented by commenting on particular decisions made. The focus of our analysis is the review output, not the participants, because we conducted linguistic analysis directly on the texts to compare the experimental groups. The language pair used in the experiments is indicated by *k *=* *1, 2, where 1 indicates English-German and 2 English-Russian. The team in the control group is built up by two human translators, *HT*_10_ and *HT*_20_, and a reviewer, *R*_10_. This control group simulates a generic form of the TRAPD process implemented by survey projects at present. The texts produced in the control group after the review meeting are compared against those produced by two treatment groups, $G_{1}^{k}$ and $G_{2}^{k}$. In the first treatment, $G_{1}^{k}$, at the T step, one translation was conducted by a human translator denoted by *HT*_31_, and the second translation was obtained when a post-editor, *PE*_11_, who received a machine-translated output, conducted full PE. The human and the post-edited translations are discussed in the team review meeting, and the process is documented in the same way as in the control group. The reviewer is denoted as *R*_21_. In a second treatment group, $G_{2}^{k}$, the first translation was also human-produced, denoted as *HT*_42_, and the second input translation for the review meeting was obtained by a post-editor using light PE, denoted as *PE*_22_. As in the other groups, there is a reviewer, denoted by *R*_32_, the translations were discussed in a team review meeting, and the process was documented. Each participant was only assigned to one group and one role.^3^ Control and treatment groups shared all other features except for the interventions defined here. The subscript *j *=* *0, 1, 2, indicates that the participant was part of the control group, the first treatment using full PE or the second treatment using light PE, respectively. In the review session, each group discussed and finalized a set of y^*k*^ translated segments. These segments correspond to translation units, such as a sentence or a response option, typically delimited by a period, a question mark, a semicolon, or a line break. Each segment is denoted by $y_{ij}^{k}$, with *i *=* *1*, …*, 268, indicating one out of 268 segments of 40 survey questions sampled from the ESS and the EVS questionnaires. In total among the two languages, we analyzed 1,608 text segments. As the focus is to compare the quality of the translations in the control groups and in the treatments, it is the text segments and not the participants which constitute our unit of analysis.

(1) $$\begin{array}{c} G_{0}^{k}\;=\;\{{\mathrm{HT}}_{10},\;{\mathrm{HT}}_{20},\;R_{10}\}\;\Leftrightarrow \;y^{k}\;=\;y_{10}^{k},\;\ldots ,\;y_{i0}^{k}\;\forall \\ \;i\;=\;1,\;\ldots ,\;268,\;j\;=\;0,\;k\;=\;1,\;2 \end{array}$$

(2) $$\begin{array}{c} G_{1}^{k}\;=\;\{{\mathrm{HT}}_{31},\;{\mathrm{PE}}_{11},\;R_{21}\}\;\Leftrightarrow \;y^{k}\;=\;y_{11}^{k},\;\ldots ,\;y_{i1}^{k}\;\forall \\ i\;=\;1,\;\ldots ,\;268,\;j\;=\;1,\;k\;=\;1,\;2 \end{array}$$

(3) $$\begin{array}{c} G_{2}^{k}\;=\;\{{\mathrm{HT}}_{42},\;{\mathrm{PE}}_{22},\;R_{32}\}\;\Leftrightarrow \;y^{k}\;=\;y_{12}^{k},\;\ldots ,\;y_{i2}^{k}\;\forall \\ i\;=\;1,\;\ldots ,\;268,\;j\;=\;2,\;k\;=\;1,\;2 \end{array}$$

### Participants

Participants had fixed role-background combinations. Human translators *HT*_10_, *HT*_31_, and *HT*_42_ were professional translators, with previous survey translation experience. Human translators *HT*_20_, post-editors *PE*_11_ and *PE*_22_, as well as reviewers *R*_10_, *R*_21_, and *R*_32_ were social scientists, with work experience in the social sciences and with experience in questionnaire design and translation. Participants had native speaker competence of either German or Russian, *k*. The combination of having both professional translators and social scientists collaborating in interdisciplinary teams mirrors how the TRAPD model is recommended to be set up in survey projects. We used snowball sampling, translator unions, as well as survey projects to recruit potential participants. Based on answers from a recruitment questionnaire, we recruited six professional translators and twelve social scientists.

We matched backgrounds and experiences to have a similar composition of teams in the control groups and in the treatments in terms of backgrounds and skills. The participants were paid for their task. Participants were informed that “the study aim is to integrate machine translation into team-based questionnaire translation procedures and to evaluate the overall process”; they were neither informed of nor requested further details of the study.^4^Supplementary Material section A provides more detailed information about the participants in the experiments. All participants, according to their roles, received virtual training and written materials on their task. Training covered, for instance, information on the implemented TRAPD model, a translation brief specifying the translation objectives (including the target group and the survey mode), do’s and don’ts in questionnaire translation, and information on the source questionnaire.

### Choice of Languages

The source questions were in the English language. German and Russian were chosen as target languages because they are used in several countries in large-scale cross-national survey projects. Both languages stand for different language families, the Proto-Germanic and the Slavic language family, respectively. As is typically the case in translation studies and linguistics, the native tongues of the research team also played a role in the language choice. By choosing German and Russian, it was possible to analyze the data ourselves, understand the contents of the review sessions, and communicate with participants and coders of translation errors.

### Instrument: Survey Questionnaire

The selection of the survey questions was done by a combination of random sampling and item selection based on criteria of known translation problems in human questionnaire translation and/or machine translation. Known translation problems included, for instance, challenging terminology; and machine translation issues included, for instance, gender issues. Supplementary Material section B provides details on the sampling of survey items, and about the criteria for the selection of items. This twofold approach ensured randomness in the selection of questions to be translated and coverage of key characteristics and potential translation challenges of a survey questionnaire.

The ESS sampling frame included questionnaires of Round 1 to Round 9 and repeated questions that were administered every round only once, adding to 1,454 questions. From the EVS, the sampling frame included Wave 1 to Wave 5, 1,745 questions in total. We sampled 262 items stratified by wave/round and study. Starting from this random sample, a final set of 40 questions, which constituted the English source of 268 segments, was selected by, first, evaluating each of the questions in the sample against the criteria and, second, ranking questions in terms of importance. A few modifications were introduced to the original source items to create a lab questionnaire. For instance, Don’t know and Refusal categories were harmonized across ESS and EVS items. Supplementary Material section C contains the source questionnaire in English, and the Supplementary Material to this article shows the source and translated questions per language and group.^5^

### Data Collection

Training of participants and data collection took place from September 15 to October 23, 2020. Participants filled in background questionnaires before, during, and after the experiments. As the experiments took place in 2020 and in-person meetings were discouraged or not legally allowed worldwide, the team-based training and review sessions were conducted online using Zoom.

### Translation and Documentation Environment

**Figure 4.**
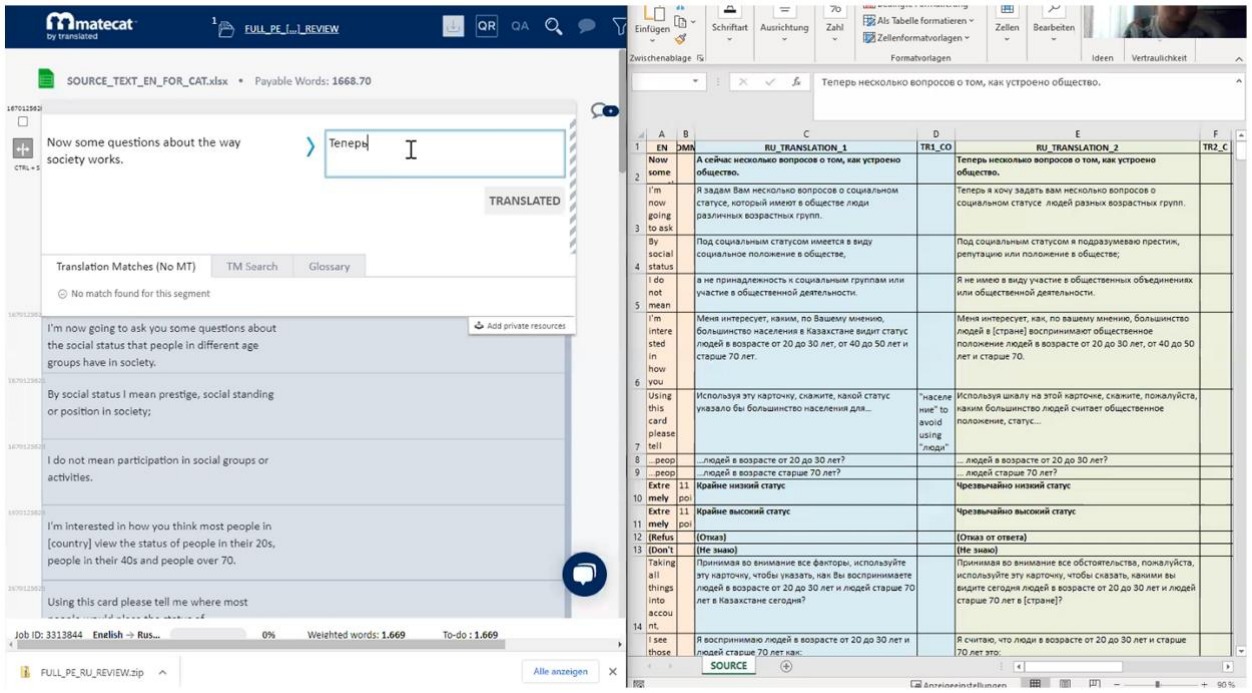
Documentation environment at the team meeting.

Depending on the role of each participant in the study, individual translation projects with and without MT features, respectively, were set up in the computer-aided translation tool MateCat (Federico et al. 2014). MateCat is open-access; it includes a neural machine translation application and PE features. The team meetings made use of the MateCat environment and spreadsheets with the translations and translator comments, as shown in figure 4.

## Analytical Approach

### Error Scheme

The error scheme used in this study is based on the harmonized MQM-DQF translation quality metrics, which combines the MQM framework^6^ and the Dynamic Quality Framework (DQF).^7^ While the MQM was set up as a comprehensive and detailed framework, drawing on many different translation metrics, DQF was based on industry best practices and focused on the issues commonly checked by language service providers (Lommel et al. 2015; Lommel 2018). We took a subset of the DQF-MQM and adapted it to the text type of survey questionnaires by selecting, omitting, or adding new subcategories.

The original four severity levels, running from critical to neutral, were adapted to three levels (major, minor, and neutral), and definitions were tailored to the survey context.

- Major errors: The translation completely changes the meaning, likely misleads the respondent, or provides incorrect, missing, and/or contradictory information.
- Minor errors: They may affect the respondent’s comprehension of translated text and increase the time required to read and to understand the translation.
- Neutral errors: They include errors that might make the translation a bit harder to understand, but ultimately do not stop the respondent from overall understanding and using the translation in terms of the measurement goal.

The final error scheme has seven categories: accuracy, fluency, survey-specific terminology/phrases and features, style, locale convention, verity, and other. Each of these categories is subdivided into subcategories that allow a fine-tuned classification of translation errors in the texts. Supplementary Material section D summarizes the error scheme definition. Every single instance of an error was coded, including repetitive errors. The comments (that is, the documentation) made by the teams in the review step were not considered during error coding to ensure an independent evaluation. The way the severity levels were defined included assessing a potential impact of erroneous wordings on respondents in a face-to-face interview. Over- or underrating the impact when choosing severity levels cannot be ruled out. However, since we applied a consistent error coding approach across all review outputs and since coders were not knowledgeable of which review version they were coding, no translation should be put at a disadvantage through the assessment.

### Translation Quality Assessment: Error Coding Process

Besides coding errors and severity levels, coders had to apply a specific error coding syntax, linking source text wording to translation errors in the target. For the error coding to achieve high quality, a harmonization approach was applied to reduce subjective or idiosyncratic language understanding (Kuckartz 2014). The setup of the approach drew on experiences from other studies (Daems, De Clercq, and Macken 2017; Koponen and Salmi 2017). Coders were provided with training for their tasks. In each language, two independent coders, not involved in the experiments, coded each of the segments included in the set, *y*, for translation errors, $z_{m(\mathrm{yij})}^{k}$. They did not know which group had produced the translation they were coding. They subsequently met with a referee in a harmonization meeting to discuss cases of diverging coding; the referee only got involved when coders could not agree on a final error coding and needed a third person for judgment. A harmonization process was preferred, as there is evidence suggesting that interrater reliability is not an appropriate method in translation quality assessment (Burchardt and Lommel 2014; Jia, Carl, and Wang 2019).^8^ During the harmonization meetings, special emphasis was placed on ensuring consistent error coding across review outputs in a language, but there was not one reference translation that would serve as a “gold standard.”

The coders were native speakers of the respective target languages, trained translators or translation practitioners, and in three out of four cases highly familiar with survey translation. The lack of familiarity with survey translation of one of the Russian coders was offset by additional training before the task and by learning-by-doing through the large number of harmonization sessions. Error coding was thus done by translation experts who were able to differentiate between necessary deviations in a translation and errors. Errors were only compared across groups within a language. We did not conduct across-language comparisons, being aware that different language pairs may have different propensities for errors.

### Statistical Analysis

The main dependent variable is defined as the count of the errors, $z_{m(\mathrm{yij})}^{k}$, where *m *=* *1, 2*, …, M* is a consecutive natural number counting the errors, and *k* and *yij* linking the error to a specific language, segment, and experimental group. The final codes after the harmonization meetings were automatically retrieved from the coding environments and the groups compared using several statistical techniques described below. Using several statistical techniques allowed us to assess the consistency of our results, and if discrepancies would be present (which is not the case), to report inconclusive results. A z-score test statistic for *H0*: *p*1 − *p*2 = 0 was defined as:

(4) $$z-\mathrm{score}\;=\;\frac{\left(\bar{p_{1}}\;-\;\bar{p_{2}}\right)\;-\;0}{\sqrt{\bar{p}\left(1\;-\;\bar{p}\right)\left(\frac{1}{n_{1}}+\frac{1}{n_{2}}\right)}}$$

where $\bar{p_{1}}$ represents the ratio of the errors in the control group,$\;z_{m(yi0)}^{k}$, divided by the total number of segments, *n*_1_ = *n*_2_ = 268; is the ratio of the errors in the treatment group, $z_{m(\mathrm{yij})}^{k}$, divided by *n*_2_. Finally, $\bar{p}$ is the total proportion of errors, calculated as the sum of the errors of the control and treatment groups divided by *n*_1_ + *n*_2_.

And, a Poisson regression is defined as

(5) $$z_{m\left(\mathrm{yij}\right)}^{k}\;∼\;\mathrm{Poisson}\left(\lambda_{m}\right)$$

(6) $$\log(\lambda )=\beta_{0}\;+\;\beta_{1x1}$$

for *m *=* *1*, …, M* where the expected count of *z_m_* is *E*(*Z*) = *λ* and where *x*_1_ is a categorical predictor with three values describing the group from which the counts of errors are estimated, among control group, full PE, and light PE. As both treatments share the same control group, multiple comparisons using z-score tests carry the risk of increasing Error Type I. Therefore, Poisson regression for the counts of errors was also estimated. For both the z-score tests and the Poisson regression, we evaluated the differences in errors for the unweighted data and the data weighted by error severity levels, *w* = *w_s_*, for severity levels, *s *=* *1, 1.5, 2, representing neutral, minor, and major error, respectively. Additionally, exploratory analysis included the use of chi-square tests defined as:

(7) $$\chi^{2}=\sum \frac{{\left(O_{m}\;-\;E_{m}\right)}^{2}}{E_{m}}$$

where *O* is the observed number of errors and *E* is the expected number of errors in each error category.

## Results

### Descriptive Analysis

Table 1 presents the total number of errors ($z^{k}$) per treatment group, the number of errors weighted by severity levels, and the mean severity level for each group. Neutral errors were assigned a weight of 1, thus they count as one error. An error considered minor was assigned a weight of 1.5, and a major error was assigned a weight of 2. The mean of the severity levels is also depicted in the table; a lower number indicates more neutral errors. As the majority of errors were of neutral severity, weighting them does not change the relative amount of errors in the groups. Although we present our tables with both languages, comparisons are strictly done within a language, that is, we only compare control and treatments within a language.

**Table 1.** Translation errors aggregated by experimental group.

Language	TRAPD set-up	Group	Number of errors ($z_{m(\text{yij})}^{k}$)	Mean error severity	Errors weighted by severity
German	Control group	$G_{0}^{1}$	36	1.31	47
German	Full PE	$G_{1}^{1}$	43	1.23	53
German	Light PE	$G_{2}^{1}$	79	1.24	98
Russian	Control group	$G_{0}^{2}$	44	1.5	66
Russian	Full PE	$G_{1}^{2}$	37	1.45	53.5
Russian	Light PE	$G_{2}^{2}$	41	1.37	56

Table 2 depicts the number of errors per error type. Treatment groups in the same language are similar to each other, except for the group using light PE in German. A plurality of errors coded for this group were errors of accuracy and they were neutral, having a severity mean of 1.17.

**Table 2.** Translation errors aggregated by category and experimental group.

Language	TRAPD set-up	Group	Category	Number of errors $\left(z_{m(\text{yij})}^{k}\right)$	Mean error severity	Errors weighted by severity
German	Control group	$G_{0}^{1}$	Accuracy	14	1.43	20.0
		$G_{0}^{1}$	Fluency	9	1.00	9.0
		$G_{0}^{1}$	Style	12	1.42	17.0
		$G_{0}^{1}$	Survey specific	1	1.00	1.0
German	Full PE	$G_{1}^{1}$	Accuracy	18	1.44	26.0
		$G_{1}^{1}$	Fluency	5	1.00	5.0
		$G_{1}^{1}$	Style	5	1.20	6.0
		$G_{1}^{1}$	Survey specific	15	1.07	16.0
German	Light PE	$G_{2}^{1}$	Accuracy	39	1.17	45.5
		$G_{2}^{1}$	Fluency	9	1.17	10.5
		$G_{2}^{1}$	Style	16	1.41	22.5
		$G_{2}^{1}$	Survey specific	15	1.30	19.5
Russian	Control group	$G_{0}^{2}$	Accuracy	20	1.75	35.0
		$G_{0}^{2}$	Fluency	7	1.29	9.0
		$G_{0}^{2}$	Style	13	1.19	15.5
		$G_{0}^{2}$	Survey specific	4	1.62	6.5
Russian	Full PE	$G_{1}^{2}$	Accuracy	13	1.54	20.0
		$G_{1}^{2}$	Fluency	3	1.33	4.0
		$G_{1}^{2}$	Style	10	1.05	10.5
		$G_{1}^{2}$	Survey specific	11	1.73	19.0
Russian	Light PE	$G_{2}^{2}$	Accuracy	12	1.54	18.5
		$G_{2}^{2}$	Fluency	12	1.25	15.0
		$G_{2}^{2}$	Style	8	1.19	9.5
		$G_{2}^{2}$	Survey specific	9	1.44	13.0

A few examples shall help to better explain the results:

$G_{0}^{1}$:

- Source: To be a good citizen, how important would you say it is for a person to…
- Target: Um ein gutter Bürger zu sein, wie wichtig ist es Ihrer Meinung nach, dass eine Person…
- Error: Over-translation—severity level neutral: It would have been more appropriate to add the female term for citizen as well, currently only the male form (“Bürger”) is used.

$G_{2}^{2}$:

- Source: Not at all
- Target: Нисколько
- Error: Scales Inconsistency—severity level minor: The word “Нисколько” is difficult to interpret out of context. The option “Вообще не выполняете” (Never do it) would be better and also fit to the question text.

$G_{2}^{1}$:

- Source: Now suppose two people from different race or ethnic groups each appear in court, charged with an identical crime they did not commit.
- Target: Nehmen wir an, zwei Menschen unterschiedlicher ethnischer Herkunft erscheinen vor Gericht und werden einer gleichen Straftat angeklagt, die sie nicht begangen haben.
- Error: Register—severity level major: “Ethnisch” on its own is difficult to understand for certain groups in society.

A *χ*^2^ test of independence was performed to examine the error categories of accuracy, fluency, style, and survey-specific errors, and the groups per language. Tables 3 and 4 summarize the *χ*^2^ test. The relation between the error categories and experimental groups was significant in the experiments using the German language, *χ*^2^ (6*, N *=* *158) = 19*, p *=* *0.004. These results are driven mainly by fewer errors than expected in the control group and a larger number of observed errors in the full-PE group.

**Table 3.** Chi-square test: observed and expected counts, English-German.

Error category	$G_{0}^{1}$	$G_{1}^{1}$	$G_{2}^{1}$	Case
Accuracy	14.00	18.00	39.00	Observed
Accuracy	16.18	19.32	35.50	Expected
Fluency	9.00	5.00	9.00	Observed
Fluency	5.24	6.26	11.50	Expected
Style	12.00	5.00	16.00	Observed
Style	7.52	8.98	16.50	Expected
Survey specific	1.00	15.00	15.00	Observed
Survey specific	7.06	8.44	15.50	Expected

*Note*: *χ*^2^ test in German language, *χ*^2^(6*, N *=* *158) = 19*, p *=* *0.004.

**Table 4.** Chi-square test: observed and expected counts, English-Russian.

Error category	$G_{0}^{2}$	$G_{1}^{2}$	$G_{2}^{2}$	Case
Accuracy	20.00	13.00	12.00	Observed
Accuracy	16.23	13.65	15.12	Expected
Fluency	7.00	3.00	12.00	Observed
Fluency	7.93	6.67	7.39	Expected
Style	13.00	10.00	8.00	Observed
Style	11.18	9.40	10.42	Expected
Survey specific	4.00	11.00	9.00	Observed
Survey specific	8.66	7.28	8.07	Expected

*Note*: *χ*^2^ test in Russian language *χ*^2^(6*, N *=* *122) = 11.96*, p *=* *0.062.

The *χ*^2^ test for the experiments using the Russian language is not significant at a *p < *0.05; nevertheless, we interpret the results as the *p*-value is just above this threshold *χ*^2^ (6*, N *=* *122) = 11.96*, p *=* *0.062. These results are driven mainly by a difference in the expected and observed survey-specific errors in the control group, and in the expected and observed fluency errors in the light-PE group. This translates into a very low number of observed survey-specific errors in the control group and a larger number of observed fluency errors in the light-PE group. However, in the Russian language experiment, these differences are not significant enough to have an effect in the z-score test or in the regression coefficients that compare the control group against the treatments.

## Main Results

We compared the control group against the treatments in two ways. First, we used z-score tests for two population proportions. This test assesses whether two groups differ significantly on some single characteristic, in this case the number of errors. A first test compared the proportion of errors in the text segments by the control group with the group using full PE. A second test compared the proportion of errors in the control group against the group using light PE. The second strategy to compare the groups was to run a Poisson regression for the counts of errors. Both z-score tests and Poisson regressions show similar and consistent results.

### Z-Score Test

Table 5 summarizes the results of the z-score tests with findings described by language below. Overall, experiments in German and Russian show positive results toward integrating the use of machine translation and full PE into TRAPD, when the review output is considered. Using MT and full PE yields equivalent results to an all-human translation setting. When integrating the use of machine translation and light PE into TRAPD, results are positive in the Russian language but negative in the German language, where the review output had a larger proportion of errors. The mean severity of the errors indicated in table 1 for this group is 1.17; it indicates that this method resulted in many neutral errors.

**Table 5.** Z-score test results.

Description	Group	*p*_1_	*p*_2_	z-score	*p*.value	CI low	CI high	Errors weighted by severity
German, full PE	$G_{1}^{1}$	0.13	0.16	0.53	0.46	−0.09	0.04	No
	$G_{1}^{1}$	0.18	0.20	0.31	0.58	−0.09	0.05	Yes
German, light PE	$G_{2}^{1}$	0.13	0.29	19.53	0.00	−0.23	−0.09	No
	$G_{2}^{1}$	0.18	0.37	23.64	0.00	−0.27	−0.11	Yes
Russian, full PE	$G_{1}^{2}$	0.16	0.14	0.52	0.47	−0.04	0.09	No
	$G_{1}^{2}$	0.25	0.20	1.42	0.23	−0.03	0.12	Yes
Russian, light PE	$G_{2}^{2}$	0.16	0.15	0.06	0.81	−0.05	0.08	No
	$G_{2}^{2}$	0.25	0.21	0.86	0.35	−0.04	0.11	Yes

*Note*: Method: two-sample test for equality of proportions with continuity correction, two-tail tests.

Confidence Interval (CI), comparisons against control groups, $G_{0}^{1}$ and $G_{0}^{2}$.

### Experiments in the German Language

The errors in the control group using only human translation are not significantly different to the errors in the group using machine translation and full PE. This result does not change when the errors are weighted by severity level. This implies that with respect to the errors in the review output, both translation outputs are of the same quality. In the case of the group using machine translation and light PE, there is a significant difference in the proportion of errors in comparison with the control group, and this difference is maintained when the errors are weighted by severity. The translation has more errors when machine translation and light PE were used.

### Experiments in the Russian Language

In the case of the experiments in Russian, the difference in errors in the group using only human translation are not statistically significant in comparison with the group using machine translation and full or light PE.

### Regression Analysis

Table 6 summarizes results of the Poisson regression models. Overall, running regression models confirms the results of the z-score tests; there is no significant impact of the group in the count of the errors in the Russian language, whereas in the German language, there is an effect of the group using machine translation and light PE.

**Table 6.** Results of the Poisson regression models.

Description	Term	Estimate	Standard error	z-value	*p*. value	CI low	CI high	Errors weighted by severity
	Intercept	3.58	0.17	21.50	0.00	3.24	3.89	No
German, full PE	$G_{1}^{1}$	0.18	0.23	0.79	0.43	−0.26	0.63	No
German, light PE	$G_{2}^{1}$	0.79	0.20	3.91	0.00	0.40	1.19	No
	Intercept	3.85	0.15	26.40	0.00	3.55	4.12	Yes
German, full PE	$G_{1}^{1}$	0.12	0.20	0.60	0.55	−0.27	0.52	Yes
German, light PE	$G_{2}^{1}$	0.73	0.18	4.14	0.00	0.39	1.09	Yes
	Intercept	3.78	0.15	25.10	0.00	3.47	4.07	No
Russian, full PE	$G_{1}^{2}$	−0.17	0.22	−0.78	0.44	−0.61	0.26	No
Russian, light PE	$G_{2}^{2}$	−0.07	0.22	−0.33	0.74	−0.50	0.36	No
	Intercept	4.19	0.12	34.04	0.00	3.94	4.42	Yes
Russian, full PE	$G_{1}^{2}$	−0.21	0.18	−1.14	0.25	−0.57	0.15	Yes
Russian, light PE	$G_{2}^{2}$	−0.16	0.18	−0.90	0.37	−0.52	0.19	Yes

### Experiments in the German Language

The expected mean of errors in the group using only human translation is significantly different to the errors in the group using machine translation and light PE, but it is not significantly different to the group using full PE. This result does not change when the errors are weighted by severity level. The incidence rate ratio is defined as follows for the unweighted data: *f* (0.79) = *e*^0^.79 = 2.20, with *s.e.* = 0.44. And for the weighted data, *f* (0.79) = *e*^0^.79 = 2.09, with *s.e.* = 0.37. The incidence rate indicates that errors occurred more often when light PE was used; by changing from the control group to this treatment, the error count is expected to increase by approximately 2 units.

### Experiments in the Russian Language

In the case of the experiments in the Russian language, the expected count of errors does not change when the group changes from control to machine translation and full PE or light PE. This means that there are no statistically significant differences in the number of translation errors in texts produced in an all-human setting in comparison to texts produced by settings that integrate machine translation and a form of PE at the initial translation step.

## Discussion and Conclusion

Over the past years, neural MT has increased the quality of MT outputs overall and has made PE more efficient. However, the quality of MT and its usability in a specific translation situation (still) depends on the text type, the available MT tool, that is, their suitability for the text type, and the language combination. With these stakes in mind, we set up a research study to assess whether the quality of the review output in the TRAPD method is affected by introducing MT and PE at the translation stage and whether these effects increase or decrease output quality. Overall, the results of this study are encouraging for the use of MT and PE within the TRAPD approach. The experiments reported here, for the German and Russian languages and for a sample of ESS and EVS survey questions, show evidence that the translation quality of the review output is hardly affected by introducing MT and PE at the initial translation stage. The effect of including MT and PE in the treatments was barely quantifiable when comparing it to the control group, which was set up to use only human translation at the initial translation stage.

The secondary research questions asked whether Russian and German translations are affected differently and if differences are conditional on the use of full or light PE. We found that the effects of integrating MT and PE into TRAPD are different between Russian and German, and yes, there are different effects depending on the type of PE used. In the Russian language, the quality of review outputs resulting from the MT and PE treatments cannot be distinguished from the quality that derives from using human translation only. In the German language, there is an increase in the number of errors when light PE is used at the initial translation stage. However, the predicted increase of such errors is 2 units.

This cannot be considered an increase of such magnitude that rules out the use of the method completely. Furthermore, the analyses, conducted both without and with weights according to error severity levels, came to the same conclusions, which is positive for the use of MT and PE when integrated into the TRAPD model. Differences between the Russian and German settings, and here in particular related to the light-PE treatment group, may be explained by different dynamics in the review discussions and/or by different quality levels of the initial translations, including the PE versions. Research taking these aspects into account is currently underway, pointing, for instance, to less extensive review discussions in the German light-PE group. In the experiments, MT and PE were implemented by participants whose background is social sciences. This shows positive evidence that MT and PE can be used in the TRAPD approach by team members whose background is not professional translation, even though at this stage we cannot conclude how post-editors perceived and performed the actual PE task.

A few notes of caution seem apt. Error counts should be put into a larger context: the review meeting should not be the final step in the translation process. In a real-life setting, there would have been room for additional proofreading beyond the context of the review discussions or the possibility to clarify (source text) issues with the developers or further colleagues. Moreover, teams would certainly have picked up further errors when implementing the questionnaire in a survey tool and testing it in quantitative and qualitative pretests (as the full TRAPD model recommends). A few reviewers’ comments point in this direction, indicating for some segments that pretesting would be useful here. Survey practitioners wishing to explore the use of MT and PE in TRAPD should take into account that the mechanisms of the effects of MT and PE can be different in other languages and settings. After all, MT quality is usually better for language combinations where MT engines have been trained on large corpora. For smaller, rarer language combinations, MT quality may be problematic (Nitzke and Hansen-Schirra 2021).

Survey practitioners should also take into consideration that this was a very controlled environment and that the team meetings were implemented with rigor. While with the research presented here, we can draw conclusions on the overall team approach and how MT and PE works within this setting, we cannot yet draw conclusions on steps prior to the review output. However, this study shows how MT may be incorporated in the TRAPD approach: always accompanied by post-editing^9^ of the raw MT outputs, preferably by full PE; with a rigorous selection of the team members, providing them with training on the workings of MT and the challenges of PE. The team should combine professional translators and social scientists. One of the translations should be done by a professional translator with survey questionnaire experience.

Importantly, the review meeting should be implemented with rigor, including the creation of comprehensive documentation. PE itself does not come without challenges; for instance, smooth wording enabled by the neural-based engines may disguise errors, which may then remain undetected. PE requires “MT literacy” to understand the working of the method and consequently to benefit from it (O’Brien and Ehrensberger-Dow 2020). The activity of PE is regarded as different from translating and thus requires different skills and consequently different training (Guerberof Arenas and Moorkens 2019). Finally, an important remark regarding data security: if online tools are used to obtain the machine translations, the questionnaire texts are shared with the MT providers; therefore, confidentiality and intellectual property should be considered.

Future research will tackle the quality of the raw machine translation output, the role of the initial translations in the review output, as well as dynamics in the different team discussions. Moreover, we encourage study replication in other language combinations. Researchers may also move one step further by testing the use of two versions using MT and PE in a review discussion and then observe the outcome compared to an all-human condition.

To sum it all up, the text outputs analyzed in this article are those considered final after the review step in the TRAPD approach. Harkness and other proponents of TRAPD argued that the review sessions in which the team discusses translation options are fundamental to translation quality. The findings of the present study point to the fact that the review meeting is a very important aspect of the TRAPD method since it seems to compensate for differences in the initial translations. The potential effect of different methods used to produce the parallel translations in the T step, in this article human translation and machine translation in combination with (full or light) PE, do not or hardly remain in the review outputs. Overall, our findings align with the trend of other text types: in some languages MT is achieving a level of maturity such that it can be considered for integration in the translation workflows. At the same time, more research is needed to understand the details of MT and PE steps themselves for the text-type questionnaire.

## Supplementary Material

nfad060_Supplementary_Data

## Data Availability

Replication data and documentation are available at https://osf.io/vre5p/?view_only=001b1d4c6ece4c45a7d7340b72dbdba1.

## Footnotes

1 A text corpus, pl., corpora, is a collection of structured texts or linguistic data.

2
Braun and Johnson (2010) provide an overview of statistical methods suitable for detecting absence of equivalence in the survey data, which may be caused by translation errors.

3 Participants in the review meetings in treatments where machine translation was used did not know which of the versions were originated by a machine translation tool. During the review meetings, the version discussed is the post-edited one, not the raw machine-translated output. Similarly, in the case of human translation, the final version created by translators is commented in the review meeting, not the first draft text at the initial translation step.

4 All participants were given information on data protection and asked for their written informed consent.

5 A repository with all the data, source texts and translations, error coding environment and commented scripts in R and Python, and additional information needed to replicate this article is available at https://osf.io/vre5p/?view_only=001b1d4c6ece4c45a7d7340b72dbdba1.

6 Developed by the EU-funded QTLaunchPad project.

7 Developed by the Translation Automation User Society (TAUS).

8 An estimate of intercoder reliability was estimated using the intraclass correlation coefficient. This statistic is available in the online repository at https://osf.io/vre5p/?view_only=001b1d4c6ece4c45a7d7340b72dbdba1.

9 General guidance on PE can be found in Massardo et al. (2016) and Nitzke and Hansen-Schirra (2021).
