# Supplementary material for: Using Machine Translation and Post-Editing in the TRAPD Approach: Effects on the Quality of Translated Survey Texts
Source: Public Opin Q. 2024 Mar 28;88(1):123–48. doi: 10.1093/poq/nfad060 (PMC11014682; doi:10.1093/poq/nfad060)
Supplement: nfad060_Supplementary_Data [file nfad060_supplementary_data.pdf]

Using Machine Translation and Post-editing in the TRA(P)D approach: effects on the quality of translated survey texts

SUPPLEMENTARY MATERIAL

Diana Zavala-Rojas[1], Dorothée Behr[2], Brita Dorer[3], Danielly Sorato[4], and Veronika Keck[5]

[1,4]RECSM - Universitat Pompeu Fabra

[1,3]European Social Survey ERIC

[2,3]GESIS - Leibniz Institute for the Social Sciences

[5]NielsenIQ

Author Note

Correspondence concerning this article should be addressed to Diana Zavala-Rojas, Department of Political and Social Sciences, RECSM, Universitat Pompeu Fabra. Ramon Trias Fargas 22-25, 08005, Barcelona, Spain. E-mail: diana.zavala@upf.edu

Using Machine Translation and Post-editing in the TRA(P)D approach: effects on the quality of translated survey texts

SUPPLEMENTARY MATERIAL

Appendix A

Profile of participants

Table A1

Key characteristics of participants

Background and experience	Social scientists (n = 12)	Professional translators (n = 6)
Sex	3 male, 9 female	2 male, 4 female
Age	average 43 years old	average 44 years old
Work experience in the social sciences	average 16 years	Not applicable (N/A)
Work experience as professional translator	N/A	average 16 years
Experience translating, reviewing or proofreading survey questionnaires	all 12 participants	all 6 participants
Experience in questionnaire development/design	12	N/A
Experience using the CAT tool MateCat	None	none

Appendix B

Items' sampling

Table B1

Distribution of the sample of questions from ESS and EVS

Study	Round	Percentage	Questions sampled
ESS	R1	9%	24
ESS	R2	7%	17
ESS	R3	4%	11
ESS	R4	4%	11
ESS	R5	5%	12
ESS	R6	4%	10
ESS	R7	3%	10
ESS	R8	2%	10
ESS	R9	2%	10
EVS	W1	11%	26
EVS	W2	22%	54
EVS	W3	9%	23
EVS	W4	11%	26
EVS	W5	7%	18
		100%	262

Table B2

Criteria for the selection of survey items

Category	Examples of sub-categories
Annotation	With/without annotation
Challenging terminology or wording	Terms related to one's feelings, state of mind, well-being (e.g., feeling depressed, lonely)
	Tricky term or wording, incl. known challenges (e.g. government, family) and technical terms that may or may not be adapted to a specific country context (e.g. unemployment benefit)
Source text issues	Ambiguous, unclear wording or item
	Linguistic distinction challenging (e.g. fair/just)
	(Strongly) idiomatic language in the source, idioms, figure of speech (e.g. worse off; hit and run)
Syntax, grammar or complexity	Response categories, items part of a longer sentence ("broken stem items")
Challenging survey or design elements	WH word (such as what, who, why) - gradation (i.e. to what extent/degree)
	Balance of request (e.g. do you agree or disagree, feeling better or worse)
	Words providing a framework for the answer (e.g., about, approximate, in general, all in all, ever, any)
	Survey-specific terminology/phrases (e.g. “card”, “Read out”; interviewer instruction: “If unclear, repeat the instructions”, “if any”)
	Fills
Known MT problems	Gender/number/unclear person (1st or 3rd person)
	Addressing persons (e.g. you)

Category	Examples of sub-categories
	Subordinate clauses with different subjects: use of pronouns, relative pronouns (i.e. which, who, whose, etc.) in second sentence (vs. noun in first sentence)

Appendix C

Source English questionnaire

Table C1

English Source Questionnaire

Study	Question text
ESS	Now some questions about the way society works.
ESS	I'm now going to ask you some questions about the social status that people in different age groups have in society.
	By social status I mean prestige, social standing or position in society;
	I do not mean participation in social groups or activities.
	I'm interested in how you think most people in [country] view the status of people in their 20s, people in their 40s and people over 70.
	Using this card please tell me where most people would place the status of...
	...people in their 20s?
ESS	...people over 70?
	Extremely low status
	Extremely high status
	(Refusal)
	(Don't know)
ESS	Taking all things into account, please use this card to say how you see people in their 20s and people over 70 in [country] today?
	I see those in their 20s and over 70 as:
	One group
	Two separate groups who are part of the same community
	Two separate groups who are not part of the same community
	Only as individuals rather than groups
	(Refusal)

Study	Question text
	(Don't know)
ESS	In the last 12 months , that is since [MONTH, YEAR], were you ever unable to get a medical consultation or the treatment you needed for any of the reasons listed on this card?<br>INTERVIEWER: Refer to the same month as the interview but of the previous year.<br>For example, if the interview takes place in September 2014, use [September 2013].<br>Could not pay for it<br>Could not take the time off work<br>Had other commitments<br>The treatment you needed was not available where you live or nearby<br>The waiting list was too long<br>There were no appointments available<br>Other reason<br>Yes<br>No<br>(Refusal)<br>(Don't know)
ESS	I am now going to ask you about the effect of social benefits and services on different areas of life in [country].<br>By social benefits and services we are thinking about things like health care, pensions and social security.<br>Using this card, please tell me to what extent you agree or disagree that social benefits and services in [country]...<br>READ OUT<br>place too great a strain on the economy?

Study	Question text
ESS	lead to a more equal society?<br>Agree strongly<br>Agree<br>Neither agree nor disagree<br>Disagree<br>Disagree strongly<br>(Refusal)<br>(Don't know)
ESS	Imagine someone in their 50s who is unemployed and looking for work.<br>This person was previously working but lost their job and is now receiving unemployment benefit.<br>What do you think should happen to this person's unemployment benefit if<br>READ OUT<br>they turn down a job because it pays a lot less than they earned previously?
ESS	they refuse to regularly carry out unpaid work in the area where they live in return for unemployment benefit?<br>This person should lose all their unemployment benefit<br>This person should lose about half of their unemployment benefit<br>This person should lose a small part of their unemployment benefit<br>This person should be able to keep all their unemployment benefit<br>(Refusal)<br>(Don't know)
EVS	Please look carefully at the following list of voluntary organisations and activities and say which, if any, do you belong to?<br>Code all mentioned.<br>Religious or church organisations

Study	Question text
	Trade unions
	Professional associations
	None
	(Refusal)
	(Don't know)
EVS	I'm going to ask how often you do certain things.
	For each activity, would you say you do them every week or nearly every week, once or twice a month, only a few times a year, or not at all?
	Interviewer: Code 'Not applicable' when respondent is not involved in work, church or club.
	Spend time with friends
	Spend time with colleagues from work or your profession outside the workplace
	Spend time with people at your church, mosque or synagogue
	Every week
	Once or twice a month
	A few times a year
	Not at all
	(Refusal)
	(Don't know)
EVS	Generally speaking, do you think that your church is/the churches are giving, in your country, adequate answers to ...
	Read out and code one answer for each.
	NB: For those belonging to a church or a religious community, ask YOUR church/ religious community.
	For those not belonging to a church or religious community ask: THE churches.
	The moral problems and needs of the individual

Study	Question text
	The problems of family life
	Yes
	No
	(Refusal)
	(Don't know)
EVS	Which, if any, of the following do you believe in?
	Read out and code one answer for each.
	God
	Sin
	Yes
	No
	(Refusal)
	(Don't know)
EVS	Which of these two statements comes closest to your own opinion?
	A: I find that both freedom and equality are important.
	But if I were to choose one or the other, I would consider personal freedom more important, that is, everyone can live in freedom and develop without hindrance.
	B: Certainly both freedom and equality are important.
	But if I were to choose one or the other, I would consider equality more important, that is, that nobody is underprivileged and that social class differences are not so strong.
	Agree with statement A
	Agree with statement B
	Neither
	(Refusal)
	(Don't know)

Study	Question text
EVS	Now I'd like you to tell me your views on various issues.<br>How would you place your views on this scale?<br>Individuals should take more responsibility for providing for themselves<br>The state should take more responsibility to ensure that everyone is provided for<br>(Refusal)<br>(Don't know)<br>On this card are three basic kinds of attitudes vis-à-vis the society we live in.<br>Please choose the one which best describes your own opinion.<br>(Code one only)<br>The entire way our society is organised must be radically changed by revolutionary action<br>Our society must be gradually improved by reforms<br>Our present society must be valiantly defended against all subversive forces<br>(Refusal)<br>(Don't know)
ESS	Using this card, please say how much you agree or disagree with the following statement.<br>Read out the statement and code in grid<br>Large differences in people's incomes are acceptable to properly reward differences in talents and efforts.<br>Agree strongly<br>Agree<br>Neither agree nor disagree<br>Disagree<br>Disagree strongly<br>(Refusal)

Study	Question text
	(Don't know)
ESS	To be a good citizen, how important would you say it is for a person to...
	READ OUT
	...support people who are worse off than themselves?
ESS	...be active in politics?
	Extremely unimportant
	Extremely important
	(Refusal)
	(Don't know)
EVS	Now I'd like you to look at this card.
	I'm going to read out some different forms of political action that people can take, and I'd like you to tell me, for each one, whether you have actually done any of these things, whether you might do it or would never, under any circumstances, do it.
EVS	Signing a petition
	Occupying buildings or factories
	Have done
	Might do
	Would never do
	(Refusal)
	(Don't know)
EVS	Can you tell me your opinion on each of the following statements?
	If someone has information that may help justice be done, generally he or she should give it to authorities
	People should stick to their own affairs and not show too much interest in what others say or do

Study	Question text
	Agree strongly<br>Agree<br>Neither agree nor disagree<br>Disagree<br>Disagree strongly<br>(Refusal)<br>(Don't know)
ESS	Now some questions about things you might have done.<br>Using this card please tell me how often you have done each of these things in the last five years?<br>How often have you...<br>READ OUT<br>...made an exaggerated or false insurance claim?<br>...committed a traffic offence like speeding or crossing a red light?<br>Never<br>Once<br>Twice<br>3 or 4 times<br>5 times or more<br>(Refusal)<br>(Don't know)
ESS	Now suppose two people from different race or ethnic groups each appear in court, charged with an identical crime they did not commit.<br>Choose an answer from this card to show who you think would be more likely to be found guilty.

Study	Question text
	The person from a different race or ethnic group than most [country] people is more likely to be found guilty
	The person from the same race or ethnic group as most [country] people is more likely to be found guilty
	They both have the same chance of being found guilty
	(Refusal)
	(Don't know)
ESS	Now look at this card.
	When you and your husband/wife/partner make decisions about the following, who generally gets their way on...
	READ OUT
	occasional more expensive purchases for the household?
	Always me
	Usually me
	About equal or both together
	Usually my spouse/ partner
	Always my spouse/ partner
	Always or usually someone else
	(Refusal)
	(Don't know)
EVS	Here is a list of things which some people think make for a successful marriage.
	Please tell me, for each one, whether you think it is very important, rather important or not very important for a successful marriage?
	(Read out each item)
EVS	Faithfulness
	An adequate income

Study	Question text
	Being willing to discuss the problems that come up between husband and wife<br>Very<br>Rather<br>Not<br>(Refusal)<br>(Don't know)
ESS	Including any time spent on maternity or parental leave, around how long in total have you spent full-time at home because you were caring for your child(ren)?<br>Please use this card.<br>No time at home full-time because of children<br>Up to six months<br>More than 6 months, up to 12 months<br>More than a year, but up to 2 years<br>More than 2 years, but up to 4 years<br>More than 4 years, but up to 10 years<br>More than 10 years<br>(Refusal)<br>(Don't know)
ESS	I will now read out a list of the ways you might have felt or behaved during the past week.<br>Using this card, please tell me how much of the time during the past week.<br>READ OUT<br>...you felt depressed?<br>...you felt that everything you did was an effort?<br>READ OUT<br>None or almost none of the time

Study	Question text
	Some of the time
	Most of the time
	All or almost all of the time
	(Refusal)
ESS	(Don't know)
	Using this card, please tell me to what extent you agree or disagree with the following statement.
	Most days I feel a sense of accomplishment from what I do.
	Agree strongly
	Agree
	Neither agree nor disagree
	Disagree
	Disagree strongly
	(Refusal)
	(Don't know)
ESS	I am going to read out a list of things about your working life.
	Using this card, please say how much the management at your work allows you...
	READ OUT
	... to be flexible in your working hours?
	...to influence your environment?
	I have no influence
	I have complete control
	(Refusal)
	(Don't know)
ESS	Using this card, please say to what extent you agree or disagree with the following statement.

Study	Question text
	Employees need strong trade unions to protect their working conditions and wages
	Agree strongly
	Agree
	Neither agree nor disagree
	Disagree
	Disagree strongly
	(Refusal)
	(Don't know)
EVS	What is your job?
	Write in and code below
	Employer/manager of establishment with 10 or more employees
	Employer/manager of establishment with less then 10 employees
	Professional worker (lawyer, accountant, teacher etc.)
	Middle level non-manual - office worker etc.
	Junior level non-manual - office worker etc.
	Foreman and supervisor
	Skilled manual worker
	Semi-skilled manual worker
	Unskilled manual worker
	Farmer: employer, manager or own account
	Agricultural worker
	Member of armed forces
	Never had a job
	(Refusal)
	(Don't know)

Appendix D
Error scheme

Table D1. Error scheme definitions

Error category<br>Error subcategory	Definition
Accuracy	The target text does not accurately reflect the source text, allowing for any differences authorized by project-specific specifications.
Addition	The target text includes text not present in the source.
Omission	Content is missing from the translation that is present in the source.
Mistranslation	The target content does not accurately represent the source content.
Over-translation	The target text is more specific than the source text.
Under-translation	The target text is less specific than the source text.
Untranslated text	Content that should have been translated has been left in the source language.
Connotations	A translation may trigger a connotation in the target audience that adds meaning in the translation that is not present in the source text and thus not wanted in the translation.
Ambiguity	The target text allows different ways of interpretation; the text has more than one meaning.
Fluency	Issues related to the form or content of a text.
Grammar	Issues related to the grammar or syntax of the text, other than spelling and punctuation.
Inconsistency	The target text shows internal inconsistency on different levels, ranging from inconsistent use of repeated survey-specific terminology or phrases over inconsistent use of core terminology to inconsistent use of more general wording.
Survey-specific terminology or phrases and features	Translation with a term or phrase other than the one expected for the domain or the specific survey conditions.
Mistranslation of survey-specific terminology or phrases	The target text does not accurately represent them as used in the target culture.
Omission of survey-specific terminology or phrases	The target text omits survey-specific terminology or phrases that would be important from a measurement or design point of view.
Addition of survey-specific terminology or phrases	The target text adds elements that are not existent in the source.
Survey mode	Specific needs of a survey mode are not taken into consideration in the translation.
Scales: Design	Design of scale not correctly rendered in the translation.
Scales: Distance and intensity	Distances between answer categories or intensity of the answer categories are not correctly rendered in translation. The intervals in the answer scale in the source language bear a specific measurement logic and should not be modified in the translation.
Scales: Grammar	Grammatical aspects (e.g., relating to genus or numerus) are not taken into consideration in the translation.
Scales: Inconsistency between question text and answer categories	A word or phrase used in both the question text and the corresponding answer scale is translated using different words.
Placeholder	Country-placeholder left in brackets or untranslated.

Appendix D
Error scheme

Table D1 (Cont.) Error scheme definitions

Error category<br>Error subcategory	Definition
Style	The target text has stylistic problems.
Awkward	The target text is written with an awkward or unidiomatic style. The content is grammatically correct, but not idiomatic.
Register	The register does not take the target population into account, in terms of their age, education and other relevant socio-demographic characteristics.
Locale convention	Translation does not adhere to locale-specific mechanical conventions and violates requirements for the presentation of content in the target culture.
Date format	The target text uses a date format inappropriate for its target culture.
Currency format	The target text uses the wrong format for currency.
Measurement format	The target text uses a measurement or number format inappropriate for its target culture.
Verity	The target text makes a statement that contradicts the reality of the target culture.
Culture-specific reference	The target text inappropriately uses a culture-specific reference that will not be understandable to the intended audience, since the referred phenomena, behaviour, etc. only exist in the source culture. An adaptation would be required, but was not implemented.
Other	Any other issues.

Intra-class correlation coefficient, English-German

This result was generated using files: *script_mt_and_pe_trapd.html* and *results_ger.csv*

Average Score Intraclass Correlation

Model: twoway

Type : consistency

Codings = 78

Raters = 2

ICC(C,2) = 0.387

F-Test, H0: r0 = 0 ; H1: r0 > 0

F(77,77) = 1.63 , p = 0.0166

95%-Confidence Interval for ICC Population Values:

0.039 < ICC < 0.609

Intra-class correlation coefficient, English-Russian

This result was generated using files: *script_mt_and_pe_trapd.html* and *results_rus.csv*

Average Score Intraclass Correlation.

Model: twoway

Type : consistency

Codings = 78

Raters = 2

ICC(C,2) = 0.625

F-Test, H0: r0 = 0 ; H1: r0 > 0

F(77,77) = 2.67 , p = 0.00013

95%-Confidence Interval for ICC Population Values:

$0.412 < ICC < 0.761$
